# Language of transducer manipulation 2.0: continuing to codify terms for effective teaching

**DOI:** 10.1186/s13089-021-00245-3

**Published:** 2021-10-28

**Authors:** Bradley End, Michael I. Prats, Joseph Minardi, Melinda Sharon, David P. Bahner, Creagh T. Boulger

**Affiliations:** 1 Department of Emergency Medicine, Robert C. Byrd Health Sciences Center, West Virginia University, 1 Medical Center Drive, PO Box 9149, Morgantown, WV 26506 USA; 2 West Virginia University School of Medicine, 64 Medical Center Drive, P.O. Box 9100, Morgantown, WV 26506-9600 USA; 3 Department of Emergency Medicine, The Ohio State University Wexner Medical Center, 750 Prior Hall, 376 W 10th Ave, Columbus, OH 43210 USA

**Keywords:** Probe motion, Ultrasound education, POCUS, Language, Transducer manipulation, Terminology

## Abstract

**Objectives:**

Accurate communication is an integral component of ultrasound education. In light of the recent global pandemic, this has become even more crucial as many have moved to virtual education out of necessity. Several studies and publications have sought to establish common terminology for cardinal ultrasound probe motions. To date, no studies have been performed to determine which of these terms have been adopted by the ultrasound community at large.

**Methods:**

A survey was developed which asked respondents to describe videos of six common probe motions in addition to providing basic demographic and training data. The survey was disseminated electronically across various academic listservs and open access resources.

**Results:**

Data were collected over a 6-week period and yielded 418 unique responses. Responses demonstrated significant variation in terminology related to all 6 cardinal probe motions. While some degree of difference in response can be accounted for by discipline of training, inter-group variation still exists in terminology to describe common probe motions. Of the survey respondents, 57.5% felt that inconsistent probe motion terminology made teaching ultrasound more difficult.

**Conclusions:**

The results demonstrate that despite efforts to codify probe motions, variation still exists between ultrasound practitioners and educators in the description of cardinal probe motions. This lack of consensus can contribute to challenges in both virtual and in-person ultrasound education.

## Introduction

The use of ultrasound within medicine has expanded rapidly into a modality that is employed by a vast array of specialties to evaluate, diagnose and monitor an ever-increasing number of pathologies [1]. As ultrasound becomes more pervasive, so too does the number of providers who engage in point-of-care ultrasonography.

As the body of ultrasound practitioners continues to grow more diverse, a number of publications have sought to define a cohesive set of terminology to describe common probe motions. The first widely distributed guideline for probe motion terminology was disseminated by the American Institute of Ultrasound in Medicine in 1999 [2]. It described five common probe motions with corresponding illustrations: slide, rock, rotate, tilt and compression. In 2005, a second technical bulletin was released by the AIUM which sought to demonstrate the previously defined terms in relation to transthoracic echocardiography [3]. Since the publication of these documents, various terms have been published in articles and texts to describe common probe motions including rocking, fanning, pitching, yawing and rolling [4–6]. An additional article published in 2016 by Bahner et al. aspired to further refine the lexicon of probe motion, using the plane of imaging and angle of insonation to the target structure to further define and refine probe motion terminology [7].

Despite various attempts by individuals, research consortia and national groups to qualify and codify common probe motion, to date, no studies currently exist which have sampled the ultrasound community at large to determine which of these terms practitioners of ultrasound commonly employ in day-to-day operation and educational environments.

## Materials and methods

**Fig. 1:**
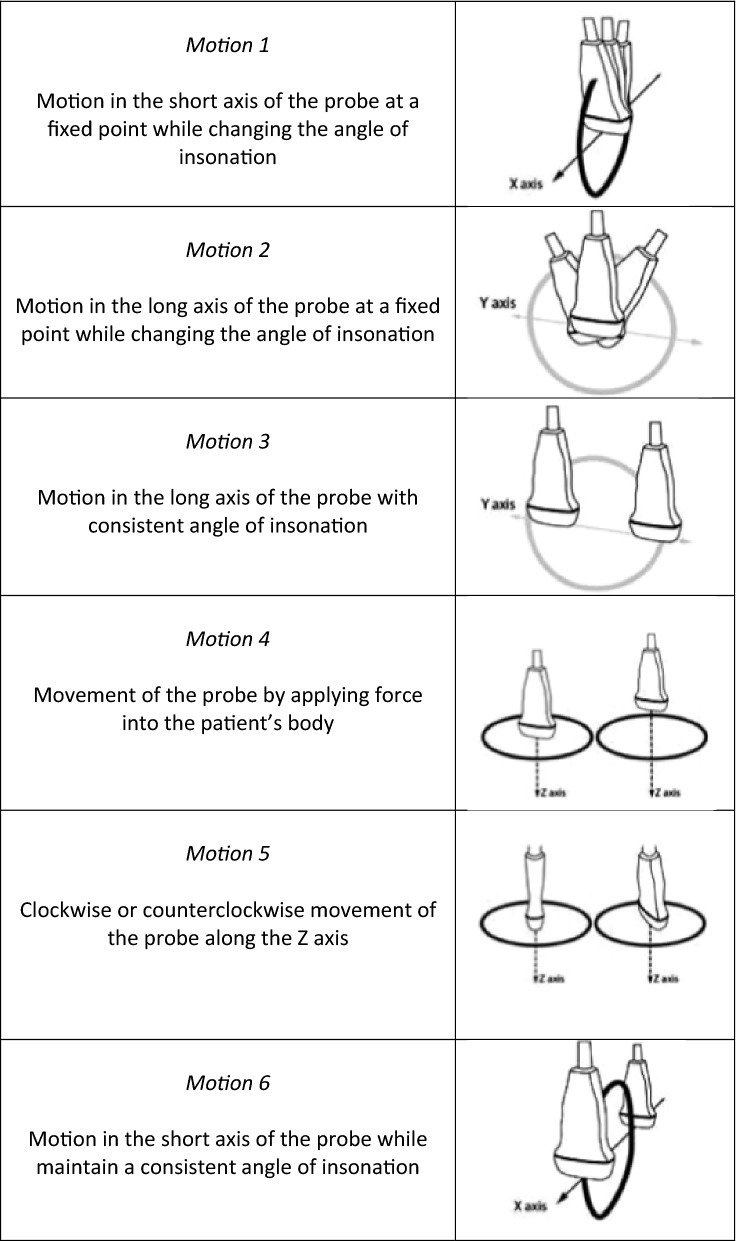
Probe motions. Images adapted from Bahner et al. Language of transducer manipulation

A 21-item survey instrument was designed by expert ultrasound educators using Qualtrics (Qualtrics, Provo, UT). The purpose of the instrument was to determine the terminology used by respondents to describe six common probe motions as shown in Fig. 1.

The instrument consisted of 6 video clips demonstrating various probe motions. Participants were able to select from a multiple choice list the term they would use to describe the probe motion shown in each video. Each item included an option for a free-text response as well. Participants also were presented with a single Likert scale survey item regarding their perception of the importance of common probe motion terminology. The remaining survey questions collected demographic data including age, gender, general training and licensure, geographic training location, practice location, and years of experience.

The survey instrument was distributed over a 6-week window. In order to collect a large number of responses from ultrasound practitioners and educators in diverse work environments, anonymous links were distributed on various ultrasound listservs, on social media and through various free, open access, medical education (FOAMed) resources. Over the course of the 6-week survey period, the link was redistributed through these media in no more than weekly intervals.

Participants who elected to participate consented to the study electronically. No identifiable information was collected and participants could withdraw from the study at any time. Participants younger than 18 years of age were excluded from the study. No compensation was provided to participants. This study was approved by the West Virginia University Institutional Review Board.

## Results

Over the course of the survey period, 418 unique surveys were returned with complete responses to the probe motion terminology segment of the instrument. Full demographic information was obtained in 74% (*n*  =  309) of surveys. These data were included in the primary analysis. Survey respondents varied in profession, practice specialty, age, years of experience, and region of training. Sixty-five percent of respondents were sonographers, 34% percent were physicians, and less than one percent were advanced practice providers. The demographics are described in detail in Table 1.

**Table 1:** Demographic data

Age	*n* = 386
18–24	30 (8%)
25–34	133 (34%)
35–44	123 (32%)
45–54	62 (16%)
55–64	32 (8%)
65–74	6 (2%)
75 +	0
Gender	*n* = 314
Male	94 (30%)
Female	218 (69%)
Other	2 (< 1%)
Training	*n* = 406
US	391 (96%)
International	15 (4%)
US training location	*n* = 377
Northeast	102 (27%)
Midwest	85 (23%)
West	49 (13%)
South	141 (37%)
Years of experience	*n* = 407
< 1	5 (1%)
1–4	88 (22%)
5–9	128 (31%)
10–14	89 (22%)
15–19	42 (10%)
20–24	20 (5%)
25 +	35 (9%)
Field of training	*n* = 411
Sonographer	269 (65%)
Physician	141 (34%)
APP	1 (< 1%)

Video clips embedded within the survey instrument demonstrated six common probe motions. Motion 1 demonstrated motion in the short axis of the probe around a fixed point while changing the angle of insonation. Thirty-seven percent (*n*  =  156) of respondents referred to this motion as “sweeping” while another 37% (*n*  =  155) of respondents referred to this motion as “fanning”. Motion 2 demonstrated motion in the long axis of the probe around a fixed point while changing the angle of insonation. Forty-six percent (*n*  =  191) referred to this motion as “rocking” and 39% (*n*  =  161) referred to this motion as “heel-toeing”. Motion 3 demonstrated motion in the long axis of the probe with a consistent angle of insonation. The majority of respondents, 88% (*n*  =  369) referred to this motion as “sliding”. Motion 4 demonstrated movement of the probe by applying force into the body of the patient. Forty-six percent (*n*  =  190) referred to this motion as “compression” while 26% (*n*  =  110) referred to this motion as “applying pressure”. Motion 5 demonstrated clockwise or counterclockwise motion of the probe along the z-axis. The majority, 61% (*n*  =  255), referred to this motion as “rotation”. Motion 6 demonstrated motion within the short axis of the probe while maintaining a consistent angle of insonation. The majority of respondents, 61% (*n*  =  255), referred to this motion as “sliding”. Table 2 shows additional responses.

**Table 2:** Probe motion terminology (*n*  =  418)

Motion 1	Sweep	Fan	Tilt	Rock	Other
Motion in the short axis of the probe at a fixed point while changing the angle of insonation	156 (37%)	155 (37%)	51 (12%)	35 (8%)	21 (5%)
Motion 2	Rock	Heel toe	Angle	Tilt	Other
Motion in the long axis of the probe at a fixed point while changing the angle of insonation	191 (46%)	161 (39%)	25 (6%)	21 (5%)	20 (5%)
Motion 3	Slide	Sweep	Drag	Advance/recede	Other
Motion in the long axis of the probe with consistent angle of insonation	369 (88%)	28 (7%)	12 (3%)	5 (1%)	4 (1%)
Motion 4	Compress	Apply pressure	Push	Press	Other
Movement of the probe by applying force into the patient’s body	190 (46%)	110 (26%)	60 (14%)	56 (13%)	2 (< 1%)
Motion 5	Rotate	Twist	Angle	Turn	Other
Clockwise or counter clockwise movement of the probe along the Z axis	255 (61%)	51 (12%)	46 (11%)	34 (8%)	32 (8%)
Motion 6	Slide	Sweep	Drag	Other	
Motion in the short axis of the probe while maintain a consistent angle of insonation	255 (61%)	116 (28%)	31 (7%)	16 (4%)	

A subgroup analysis revealed differences in the responses of sonographers and physicians (Table 3). Fifty-five percent (*n*  =  147) of sonographers referred to Motion 1 as “sweeping” while 84% (*n*  =  119) of physician respondents referred to this motion as “fanning”. For Motion 2, 69% (*n* =   97) of physicians referred to this motion as “rocking” and 55% (*n*  =  147) of sonographers referred to this motion as “heel-toeing”. For other motions there was much more consistency between fields. The majority of physicians and sonographers referred to Motion 3 as “sliding” (92% *n*  =  130 and 87% *n*  =  233, respectively). Regarding Motion 4, no clear majority was evident in sonographers’ responses, while half of physicians (50% *n*  =  70) referred to this motion as “compression”. A near majority of sonographers (48%, *n*  =  130) referred to Motion 5 as “rotation”, as did the majority of physician respondents (85%, *n*  =  120). Both the majority of sonographers and physicians referred to Motion 6 as “sliding” (61%, *n*  =  164 and 62%, *n*  =  87, respectively).

**Table 3:** Comparison of sonographer and physician responses

Motion 1	Sweep	Fan	Tilt	Rock	Other
Physician	6 (4%)	119 (84%)	12 (9%)	3 (2%)	1 (1%)
*n* = 141					
Sonographer	147 (55%)	33 (12%)	38 (14%)	31 (12%)	20 (7%)
*n* = 269					
Motion 2	Rock	Heel–toe	Angle	Tilt	Other
Physician	97 (69%)	10 (7%)	12 (9%)	14 (10%)	8 (6%)
Sonographer	91 (34%)	147 (55%)	13 (5%)	7 (3%)	11 (4%)
Motion 3	Slide	Sweep	Drag	Advance/recede	Other
Physician	130 (92%)	2 (1%)	5 (4%)	1 (1%)	3 (2%)
Sonographer	233 (87%)	25 (9%)	7 (2.6%)	3 (1%)	1 (< 1%)
Motion 4	Compress	Apply pressure	Push	Press	Other
Physician	70 (50%)	38 (27%)	13 (9%)	18 (13%)	2 (1%)
Sonographer	113 (42%)	72 (27%)	46 (17%)	38 (14%)	0 (0%)
Motion 5	Rotate	Twist	Angle	Turn	Other
Physician	120 (85%)	9 (6%)	7 (5%)	1 (1%)	4 (3%)
Sonographer	130 (48%)	42 (16%)	36 (13%)	33 (12%)	28 (10%)
Motion 6	Slide	Sweep	Drag	Other	
Physician	87 (62%)	33 (23%)	13 (9%)	8 (6%)	
Sonographer	164 (61%)	81 (30%)	17 (6%)	7 (3%)	

Survey participants were asked if they felt that inconsistent probe motion terminology made teaching more difficult. A majority of respondents either agreed (48.5%, *n*  =  185) or strongly agreed (9%, *n*  =  34) with this statement.

## Discussion

Ultrasound education is rapidly expanding across medical specialties and a variety of medical providers [8, 9]. As the number of providers interfacing with ultrasound increases, so does the publication of various technical bulletins, articles and texts related to ultrasound, however, despite this growth and dissemination of point-of-care ultrasound education, no studies have previously been performed to assess for consistency in transducer manipulation terminology. Our study is the first to attempt to qualify differences in probe motion terminology while evaluating subgroups that account for commonality as well as divergence of this terminology.

Probe motion terminology appeared to be more dependent on practice profession than any other demographic and educational factors. Considering that sonographers and physicians are predominantly trained in separate and distinct academic programs, it is not surprising that differences in terminology exist between these two groups.

This study was subject to a number of limitations. Given the size and diversity of the ultrasound community, convenience and snowball sampling techniques were utilized in an attempt to reach as many within the community as possible. This method and the anonymity of the survey does allow for breadth of distribution, but limits the ability to calculate response rates. Convenience and snowball sampling techniques are also susceptible to response bias as the captured population may not be representative of the ultrasound community at large. Despite attempts at widespread distribution, the overall response rate for this survey was likely low compared to the total population of ultrasound users. There is evidence of a skewed regional response rate in addition to a lack of international response. The vast majority of physician respondents were trained in Emergency Medicine, thus it is difficult to postulate if additional variations in terminology exist between medical specialties.

At this time, there exists no clear consensus in regard to the universal nomenclature used for various probe motions. These variations in terminology do not appear to be due to differences in age, years of experience or region. Ultimately, a multi-disciplinary consensus on the language of transducer manipulation would benefit the users and educators of ultrasound. This is especially prudent as time and distance make virtual learning and tele-health increasingly prevalent. A codified terminology agreed upon by practitioners and educators across disciplines, specialties, and geographical locations across the globe would aid both instructors and learners by allowing for clear descriptions when guiding an ultrasound exam remotely without touching the transducer. Regarding the current landscape of terminology, both physicians and sonographers generally agree that a lack of cohesive lexicon makes teaching ultrasound more difficult. Future studies should seek to include a broader audience both domestically and internationally to determine if additional factors affect differences in transducer manipulation terminology.

## Data Availability

The datasets used and/or analyzed during the current study are available from the corresponding author on reasonable request.
